# Classification of atrophic mucosal patterns on Blue LASER Imaging for endoscopic diagnosis of *Helicobacter pylori*-related gastritis: A retrospective, observational study

**DOI:** 10.1371/journal.pone.0193197

**Published:** 2018-03-29

**Authors:** Yoshiyuki Nishikawa, Yoshio Ikeda, Hidehiro Murakami, Shin-ichiro Hori, Kaori Hino, Chise Sasaki, Megumi Nishikawa

**Affiliations:** 1 Nishikawa Gastrointestinal Clinic, Matsuyama, Ehime, Japan; 2 Endoscopy Center, Ehime University Hospital, Toon, Ehime, Japan; 3 Department of Internal Medicine, Saiseikai Matsuyama Hospital, Matsuyama, Ehime, Japan; 4 Department of Gastroenterology, National Hospital Organization Shikoku Cancer Center, Matsuyama, Ehime, Japan; University Hospital Llandough, UNITED KINGDOM

## Abstract

**Background:**

Atrophic gastritis can be classified according to characteristic mucosal patterns observed by Blue LASER Imaging (BLI) in a medium-range to distant view.

**Aims:**

To facilitate the endoscopic diagnosis of *Helicobacter pylori* (HP)-related gastritis, we investigated whether atrophic mucosal patterns correlated with HP infection based on the image interpretations of three endoscopists blinded to clinical features.

**Methods:**

This study included 441 patients diagnosed as having atrophic gastritis by upper gastrointestinal endoscopy at Nishikawa Gastrointestinal Clinic between April 1, 2015 and March 31, 2016. The presence/absence of HP infection was not taken into consideration. Endoscopy was performed using a Fujifilm EG-L580NW scope. Atrophic mucosal patterns observed by BLI were classified into Spotty, Cracked and Mottled. Image interpretation results were that 89, 122 and 228 patients had the Spotty, Cracked and Mottled patterns, respectively, and 2 patients an undetermined pattern. Further analyses were performed on 439 patients, excluding the 2 with undetermined patterns.

**Results:**

The numbers of patients testing negative/positive for HP infection in the Spotty, Cracked and Mottled pattern groups were 12/77, 105/17, and 138/90, respectively. The specificity, positive predictive value and positive likelihood ratio for endoscopic diagnosis with positive HP infection based on the Spotty pattern were 95.3%, 86.5% and 8.9, respectively. In all patients with the Spotty pattern before HP eradication, the Cracked pattern was observed on subsequent post-eradication endoscopy.

**Conclusions:**

The Spotty pattern may represent the presence of HP infection, the Cracked pattern, a post-inflammatory change as seen after HP eradication, and the Mottled pattern, intestinal metaplasia.

## Introduction

Persistent infection with *Helicobacter pylori* (HP) is known to promote chronic atrophic gastritis [1–3]. Furthermore, histological gastritis resulting from HP infection can be cured by bacterial eradication [4]. In HP-related gastritis, however, atrophic changes may show amelioration, but not complete resolution endoscopically, after bacterial eradication [5]. It is thus difficult to determine whether or not bacteria have been eradicated based only on the presence of atrophic gastritis. Cumulative findings have enabled endoscopists to determine the presence or absence of HP infection. The ability to endoscopically capture pathological changes of atrophic gastritis, including the presence or absence of HP infection, facilitates more detailed assessment of progression, regression, localization and other behaviors of HP-related gastritis.

Blue LASER Imaging (BLI) [6,7] is an endoscopic technique that allows for narrow-band light observation through the use of a short-wavelength laser. This technique was initially recognized as a useful option for examining superficial blood vessels and mucosal surface structures. When observed by BLI in intermediate to distant views, atrophic gastritis appears white and brown with more intensified contrast than that observed with conventional white light. Although white light has also been applied to endoscopic observation of atrophic changes involving the gastric mucosa [8], observation by BLI facilitates determination of atrophic changes more effectively than white light. In this way, characteristic patterns of atrophic gastric mucosa on BLI can be identified, and further classified into several types.

A previous pilot study on atrophic mucosal patterns determined by an attending endoscopist, who had performed the endoscopic examinations with a detailed understanding of the patients’ conditions, suggested associations between atrophic mucosal patterns and HP infection. Therefore, in order to verify these results, we conducted a retrospective observational study by having three endoscopists, blinded to the clinical features of the patients, re-evaluate the atrophic mucosal patterns of those included in the pilot study. The objectives of the current study were to determine the implications of each atrophic mucosal pattern and clarify the associations between each of these pattern and HP infection, thereby demonstrating the usefulness of classifying atrophic mucosal patterns as a means of facilitating the endoscopic diagnosis of HP-related gastritis.

## Methods

### Participants and study methods

A total of 1140 patients underwent upper gastrointestinal (GI) endoscopy at Nishikawa Gastrointestinal Clinic during the 1-year period from April 1, 2015 to March 31, 2016. Among them, we included in this study 441 patients who were found to have atrophic gastritis by endoscopic observation using white light, and in whom the presence/absence of HP infection was confirmed, based on the examination data. For patients who underwent multiple endoscopy sessions during the enrollment period, only the data from the first session were included, i.e. those from the subsequent sessions were excluded. Additional data of patients who underwent GI endoscopy after April 1, 2016 were also included in the analysis of histopathological findings in this study. Patients who underwent HP eradication and subsequent post-eradication endoscopy were examined for changes in atrophic mucosal patterns after versus before eradication.

Endoscopy was performed with an EG-L580NW nasal upper GI endoscope (FUJIFILM Medical Co. Ltd., Tokyo, Japan) inserted either orally or nasally. For BLI, the bright mode was used such that bright images could be obtained in an intermediate or distant view.

This study was approved by the Ethics Committee of Ehime University Hospital (Approval No. 1605010).

### Classification of patterns

**Fig 1:**
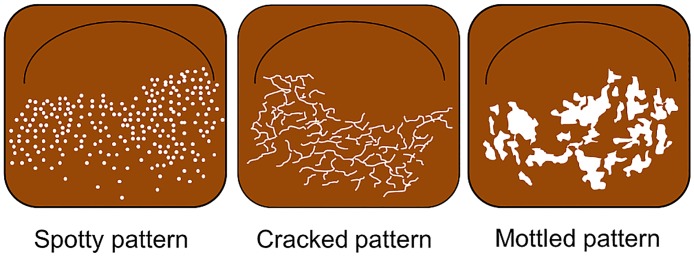
Illustrations of the Spotty, Cracked and Mottled patterns.

Fig 1 is an illustrative chart of the Spotty, Cracked and Mottled patterns.

**Fig 2:**
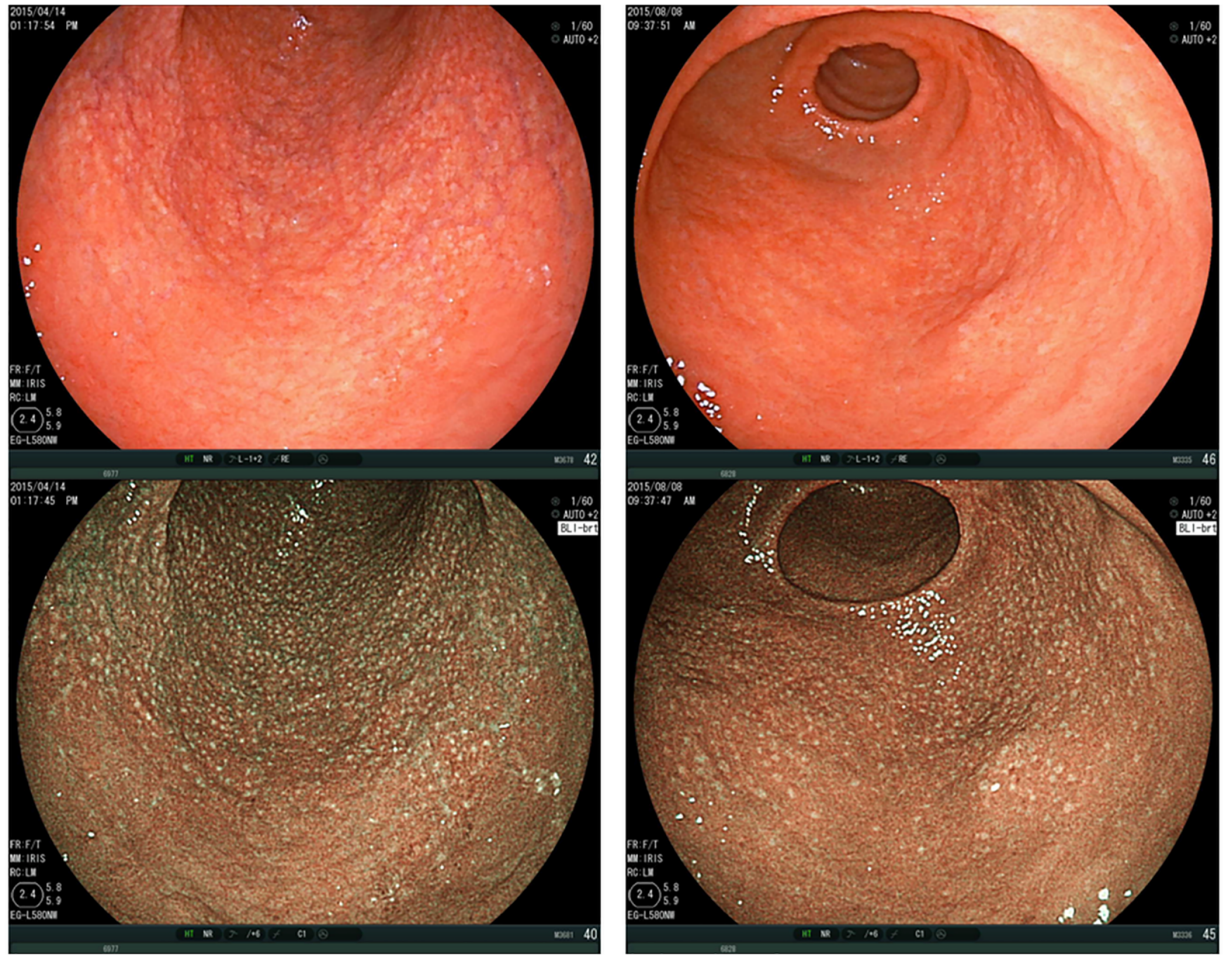
Spotty pattern. Gastric antrum, observed by BLI-bright mode, shows a typical “Spotty pattern”. Upper panels: Images taken under white light. Lower panels: Images observed by BLI. BLI, Blue LASER Imaging.

**Fig 3:**
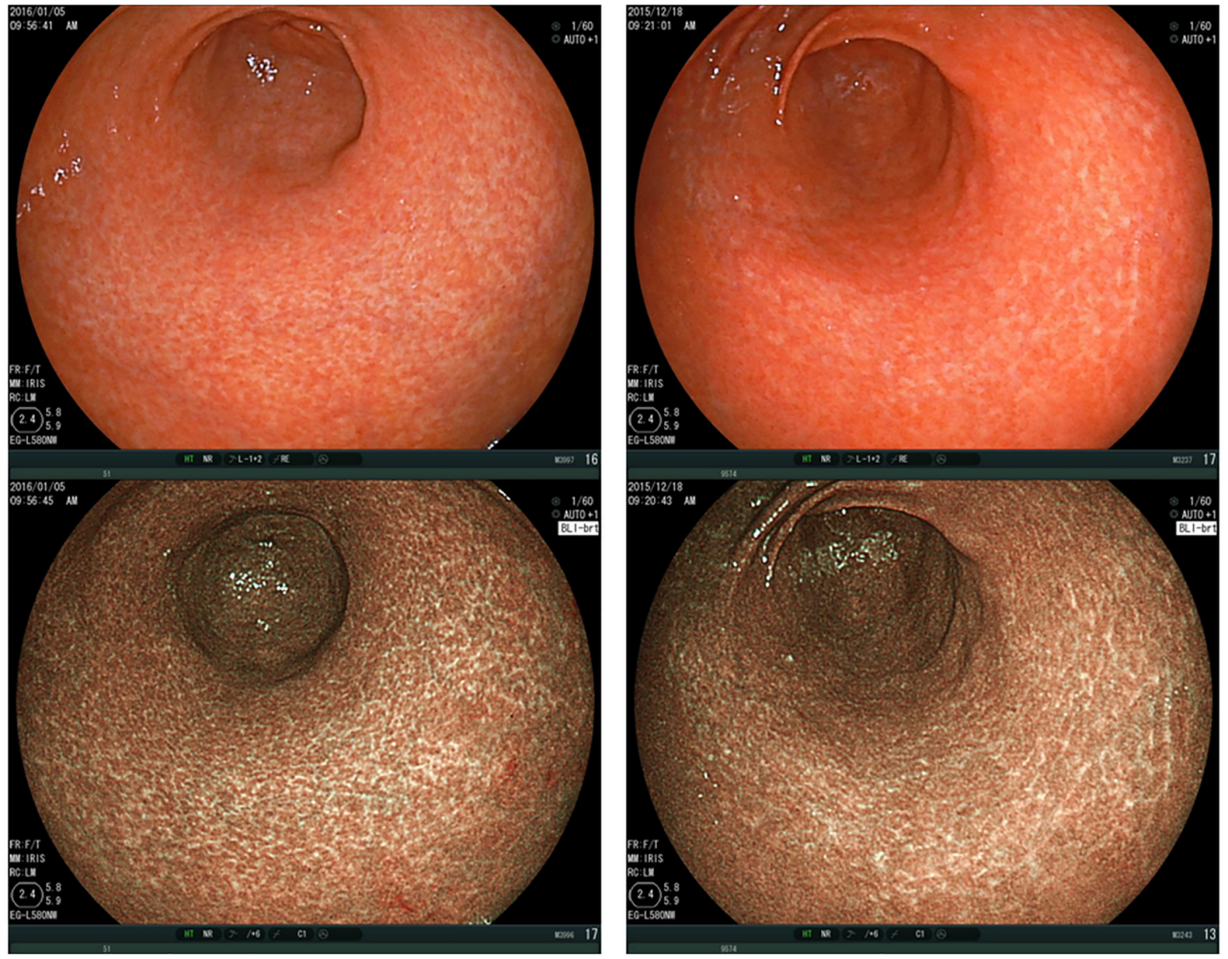
Cracked pattern. Gastric antrum, observed by BLI-bright mode, shows a typical “Cracked pattern”. Upper panels: Images taken under white light. Lower panels: Images observed by BLI. BLI, Blue LASER Imaging.

**Fig 4:**
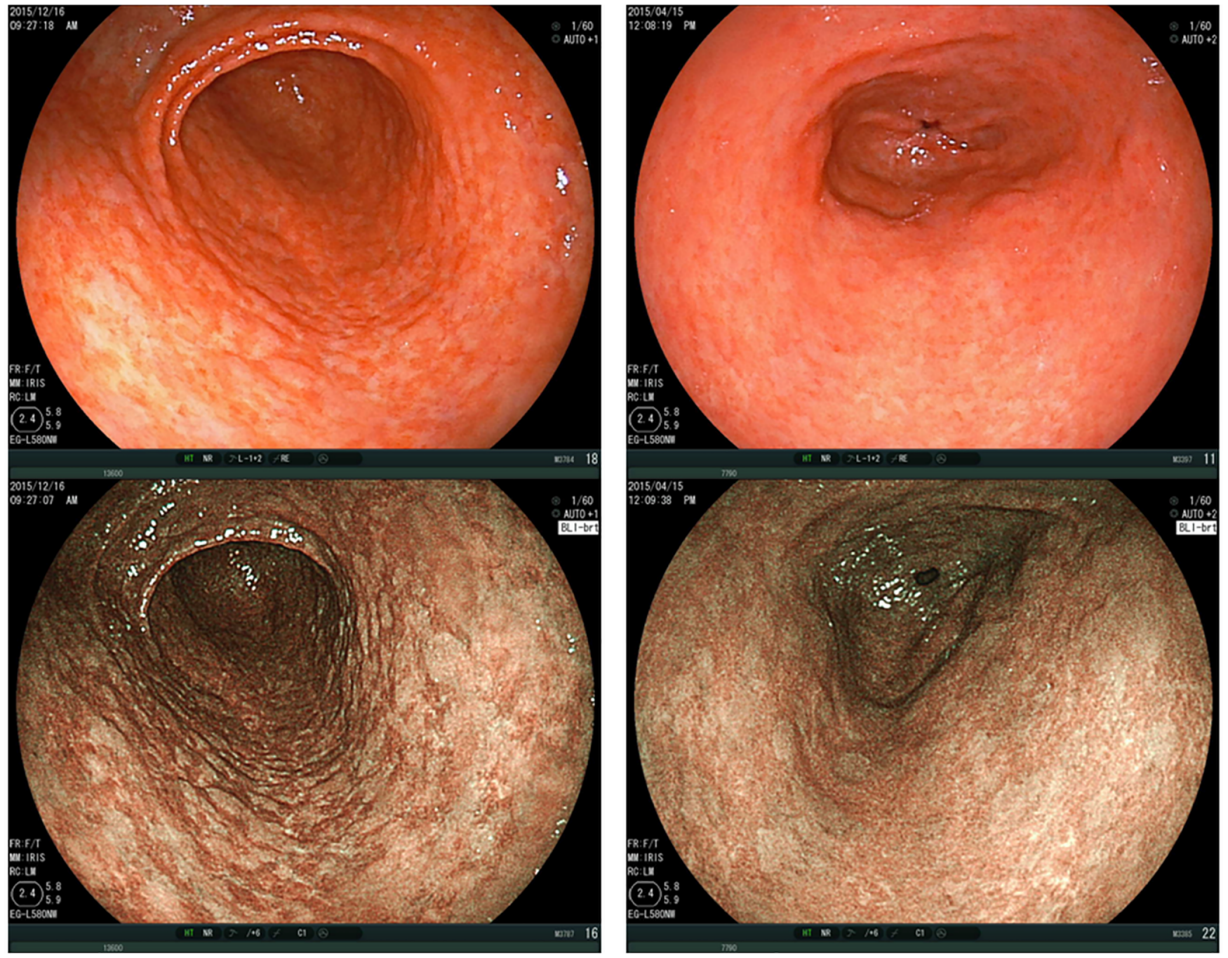
Mottled pattern. Gastric antrum, observed by BLI-bright mode, shows a typical “Mottled pattern”. Upper panels: Images taken under white light. Lower panels: Images observed by BLI. BLI, Blue LASER Imaging.

The patterns of atrophic gastric mucosa observed by BLI were classified as follows:

1. Spotty pattern (Fig 2)
  This is an atrophic mucosal pattern with a spotty appearance. Under BLI, this type of atrophy was observed as a spotty pattern consisting of white spots 1–2 mm in diameter. The isolated spots were considered to represent the Spotty pattern. Scarce, scattered spots were not considered to constitute this pattern. At least a few spots, forming an aggregate, had to be identifiable for a designation of the Spotty pattern.
2. Cracked pattern (Fig 3)
  This is an atrophic mucosal pattern with a cracked appearance. This pattern was observed as white net-like cracks, consisting of lines. The cracks varied from narrow to wide. The pattern consisting of white cracks, with widths equal to or greater than those in the brown areas, was classified as the Mottled pattern as described below.
3. Mottled pattern (Fig 4)
  This is an atrophic mucosal pattern with a mottled appearance. The whitish area varies from a narrow/patchy to a wide/mottling pattern. This pattern includes a wide range of findings not classifiable as either the Spotty or the Cracked pattern.

When more than one pattern was observed in a patient, that adjacent to the atrophic border on the greater curvature side [7] was regarded as the representative atrophic mucosal pattern. Therefore, in many cases, atrophic gastritis by BLI was evaluated in the greater curvature of the antrum. The patients with Spotty-pattern atrophy in any part of the stomach, even if the spotty-patterned area was limited, were considered to have the Spotty pattern.

### Determination of patterns

Atrophic mucosal patterns were determined as follows:

1. Three GI endoscopists from medical institutions other than Nishikawa Gastrointestinal Clinic, who each had at least 20 years (24, 23, 23 years) of endoscopic experience and were blinded to patients’ clinical information, such as the presence or absence of HP infection, independently interpreted the endoscopic images to classify the atrophic mucosal findings into the Spotty, Cracked, Mottled, or “Undetermined” pattern.
2. When a pattern determination was agreed upon by all three endoscopists, that pattern was adopted.
3. When a pattern determination was agreed upon by two endoscopists but not by the other, the pattern agreed upon by the two endoscopists was adopted.
4. When the three endoscopists all had different pattern determinations, the final determination was made by discussing the findings until consensus was reached.
5. The anonymized image data only were provided to the interpreting endoscopists, without other clinical information.
6. To determine whether the current mucosal pattern classification can be made by non-experts, we asked three non-expert endoscopists (with 4, 2, and 1 year of endoscopic experience) to interpret the same endoscopic images. We then evaluated the interpretation agreement rate between the three experts and the three non-experts.

The presence or absence of HP infection was determined as follows. The use of examination data obtained at other medical institutions was allowed for evaluation only if the data were confirmed to include those pertaining to HP infection. HP infection was diagnosed based on anti-HP immunoglobulin G (IgG) antibody serology [9,10], the urea breath test [11–14] and biopsy. Anti-HP IgG antibody titer < 3 was defined as negative, ≥ 10 as positive, and 3–9 as those requiring additional tests for confirmation.

Associations between the atrophic mucosal pattern and age, sex, presence/absence of HP infection and history of HP eradication were evaluated.

### Statistical analysis

Student’s t-test was used for analysis of the age variable, and the Chi-squared test was used for analyses of the other items shown in Table 1. A *P* value < 0.05 was considered to indicate a statistically significant difference.

**Table 1:** Background characteristics of patients with atrophic gastritis on BLI.

Atrophic mucosal pattern	Total		Spotty (S)	Cracked (C)	Mottled (M)	*P* value
**Number of patients**	439		89	122	228	–
**Age (years), mean (± SD)**	65.6 (± 13.3)		58.5 (± 16.0)	64.7 (± 11.6)	68.9 (± 11.8)	< 0.001 (S vs C)*
						< 0.0001 (S vs M)*
						< 0.001 (C vs M)*
**Sex**	Female	279	65	74	137	0.061 (S vs C)
	Male	160	24	48	91	0.031 (S vs M) *
						0.918 (C vs M)
**HP infection**	Negative	255	12	105	138	< 0.001 (S vs C)*
	Positive	184	77	17	90	< 0.001 (S vs M)*
						< 0.001 (C vs M)*
**History of HP eradication**	Yes	243	79	37	127	< 0.001 (S vs C)*
	No	196	10	85	101	< 0.001 (S vs M)*
						< 0.001 (C vs M)*

BLI, Blue LASER Imaging; SD, standard deviation; HP, *Helicobacter pylori*.

*P < 0.05.

### Histopathological examination

Histopathological examinations were performed using biopsy specimens collected from the patients who underwent biopsy for diagnosis of HP infection. Therefore, only a limited number of patients were included in the histopathological assessment.

## Results

### Image interpretation by endoscopists

Image interpretations were agreed upon by three endoscopists for 195 patients, by two endoscopists for 221 patients, and the three endoscopists disagreed regarding 25 patients. For the 25 patients, the image data were re-evaluated by the three endoscopists and a consensus was reached for pattern determination. This re-evaluation resulted in 89, 122 and 228 patients being deemed to have the Spotty, Cracked and Mottled patterns, respectively, and 2 patients an undetermined pattern. Further analyses were performed on 439 patients, excluding the 2 patients with undetermined patterns.

The agreement rate among the three endoscopists was evaluated for each pattern. The numbers of patients, in whom pattern classification was agreed upon by all three endoscopists/by two of the endoscopists/by none of the three endoscopists, were 44 (49.4%)/40 (44.9%)/5 (5.6%) for the Spotty pattern, 22 (18.0%)/84 (68.9%)/16 (13.1%) for the Cracked pattern, and 129 (56.6%)/95 (41.7%)/4 (1.8%) for the Mottled pattern. The numbers of patients in whom pattern classification was divided between the Spotty and Cracked patterns, the Spotty and Mottled patterns, the Cracked and Mottled patterns, and undetermined patterns, were 13, 41, 154, and 13, respectively, indicating that the Cracked pattern was associated with a low agreement rate and tended to be difficult to differentiate from the Mottled pattern. The kappa statistic was used to analyze interobserver agreement among endoscopists A, B and C, who interpreted the endoscopic findings independently of each other. The kappa coefficient between A and B was 0.54, indicating a moderate agreement rate, while those between A and C and between B and C were 0.34 and 0.16, respectively, indicating insufficient agreement rates. The number of patients classified as having the Spotty/Cracked/Mottled/undetermined patterns by endoscopists A, B and C were 88/148/202/3, 106/161/159/15 and 57/33/350/1, respectively, suggesting that endoscopist C tended to classify fewer patients as having the Cracked pattern and more patients as having the Mottled pattern. The Spotty pattern was associated with a relatively high degree of interobserver agreement. The agreement rate might have been low because this was the first experience for these endoscopists to interpret such endoscopic findings. Interpretation by endoscopists with further training and experience in this field is anticipated to achieve a higher agreement rate in future studies.

When the agreement rates among endoscopists were evaluated separately for the Spotty pattern and the other two patterns, the kappa coefficient was 0.74 between endoscopists A and B, 0.58 between A and C, and 0.51 between B and C, suggesting that the Spotty pattern can be identified without difficulty.

We then investigated whether the three non-expert endoscopists (endoscopists D, E and F) were able to correctly classify these patterns through interpretation of the same images. The kappa coefficients between D and E, E and F and D and F were 0.55, 0.55 and 0.48, respectively, indicating a moderate agreement rate. We also investigated the agreement rate for the overall judgments made by expert and non-expert endoscopists using the same protocol, and obtained a kappa coefficient of 0.74, indicating a substantial agreement rate.

The mean age of the 439 patients evaluated was 65.6 ± 13.3 years, with a female/male ratio of 279:160. Two-hundred fifty-five (255) patients tested negative and 184 positive for HP infection. Of these, 196 patients had a history of HP eradication and 243 did not.

### Analysis of atrophic mucosal patterns

We then performed the analysis according to atrophic mucosal pattern. The mean ages of the patients with the Spotty, Cracked and Mottled patterns were 58.5 ± 16.0, 64.7 ± 11.6 and 68.9 ± 11.8 years, respectively, with significant differences between the Spotty and Cracked patterns (*P* < 0.001), between the Spotty and Mottled patterns (*P* < 0.0001), and between the Cracked and Mottled patterns (*P* < 0.001, Student’s t-test).

As for the sex difference, the Spotty pattern was observed more frequently in women than in men, as revealed by the comparison between the Spotty and Mottled patterns (*P* = 0.031, Chi-squared test). For other patterns, no significant tendencies were observed.

### Relationships between atrophic mucosal patterns and HP infection

We then examined the relationships between the atrophic mucosal patterns and HP infection. Twelve patients with the Spotty pattern tested negative, while the majority (77 patients) tested positive for HP. All 12 patients with the Spotty pattern who tested negative for HP were female. Among those with the Cracked pattern, the majority (105 patients) tested negative, while 17 patients tested positive. Of the patients with the Mottled pattern, 138 tested negative and 90 positive for HP.

As for the relationships with a history of HP eradication, the majority (79) of patients with the Spotty pattern had not undergone bacterial eradication, with only 10 patients having a history of eradication. Among the patients with the Cracked pattern, 85 had and 37 had not undergone bacterial eradication. The patients with the Mottled pattern included 101 patients with and 127 without a history of bacterial eradication (Table 1).

We further examined the relationship between HP infection status (negative/positive) and a history of bacterial eradication. In the entire population, 255 patients who tested negative for HP infection included 187 patients with and 68 without a history of bacterial eradication, while 184 patients who tested positive for HP infection included 175 patients without prior bacterial eradication and 9 in whom prior eradication had failed.

**Fig 5:**
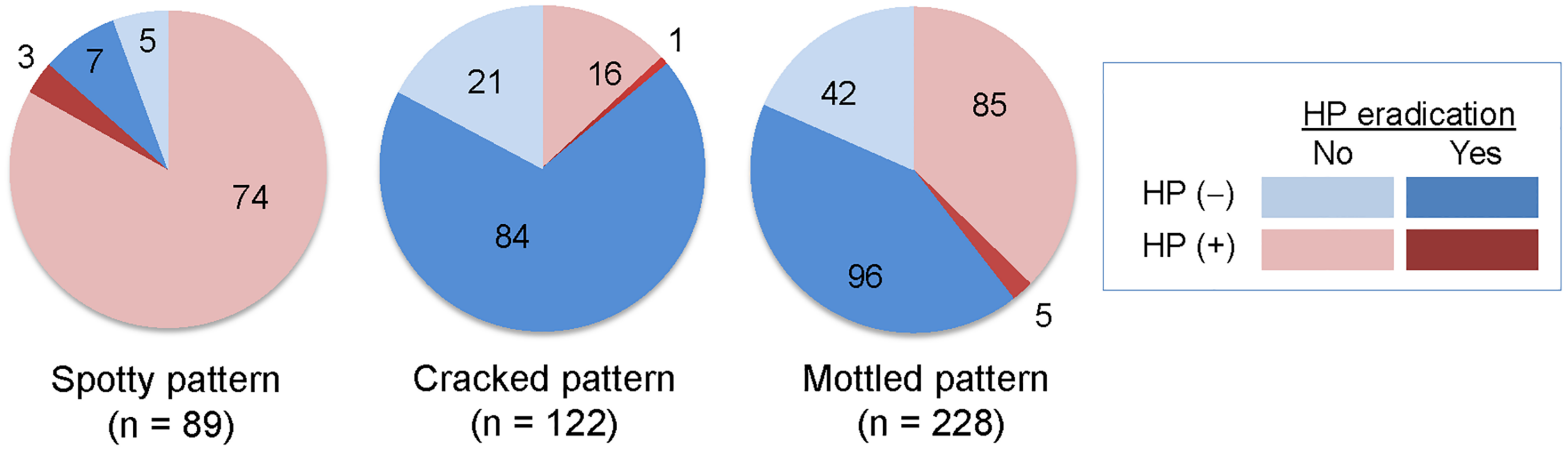
Relationships between atrophic mucosal pattern and HP infection. Numbers of patients with presence or absence of HP infection and history of HP eradication. HP, *Helicobacter pylori*.

Among the 89 patients with the Spotty pattern, 12 tested negative for HP infection, of whom 7 had and 5 had not undergone bacterial eradication. Of the remaining 77 patients who tested positive for HP infection, 74 had not undergone bacterial eradication, while eradication had failed in the other 3 patients. Among the 122 patients with the Cracked pattern, 105 tested negative for HP infection, of whom 84 had and 21 had not undergone bacterial eradication. Of the remaining 17 patients who tested positive for HP infection, 16 had not undergone eradication while therapy had failed in the other. The 228 patients with the Mottled pattern included 138 patients who tested negative for HP infection, of whom 96 had and 42 had not undergone bacterial eradication. Of the remaining 90 patients who tested positive for HP infection, 85 had not undergone bacterial eradication, while therapy had failed in the other 5 (Fig 5).

It may be assumed that the Spotty pattern indicates the endoscopic finding of HP positivity. Namely, those with the Spotty pattern (“Present”) consisted of 77 patients who tested positive and 12 who tested negative for HP infection, while those without the Spotty pattern (“Absent”) consisted of 107 positive and 243 negative patients. The sensitivity, specificity, positive predictive value and negative predictive value (95% confidence interval) were calculated to be 41.8% (38.1–44.5%), 95.3% (92.6–97.2%), 86.5% (78.7–91.9%) and 69.4% (67.5–70.8%), respectively. The positive likelihood ratio was 8.9 (5.1–15.8).

Similarly, it may be assumed that the Cracked pattern indicates the endoscopic finding of HP negativity. Namely, those with the Cracked pattern (“Present”) consisted of 105 patients who tested negative and 17 who tested positive for HP infection, while those without the Cracked pattern (“Absent”) consisted of 150 negative and 167 positive patients. The sensitivity, specificity, positive predictive value and negative predictive value were calculated to be 41.2% (38.1–43.5%), 90.8% (86.5–93.9%), 86.1% (79.7–90.9%) and 52.7% (50.2–54.5%), respectively. The positive likelihood ratio was 4.5 (2.8–7.2). Values are rounded off to one decimal place.

**Fig 6:**
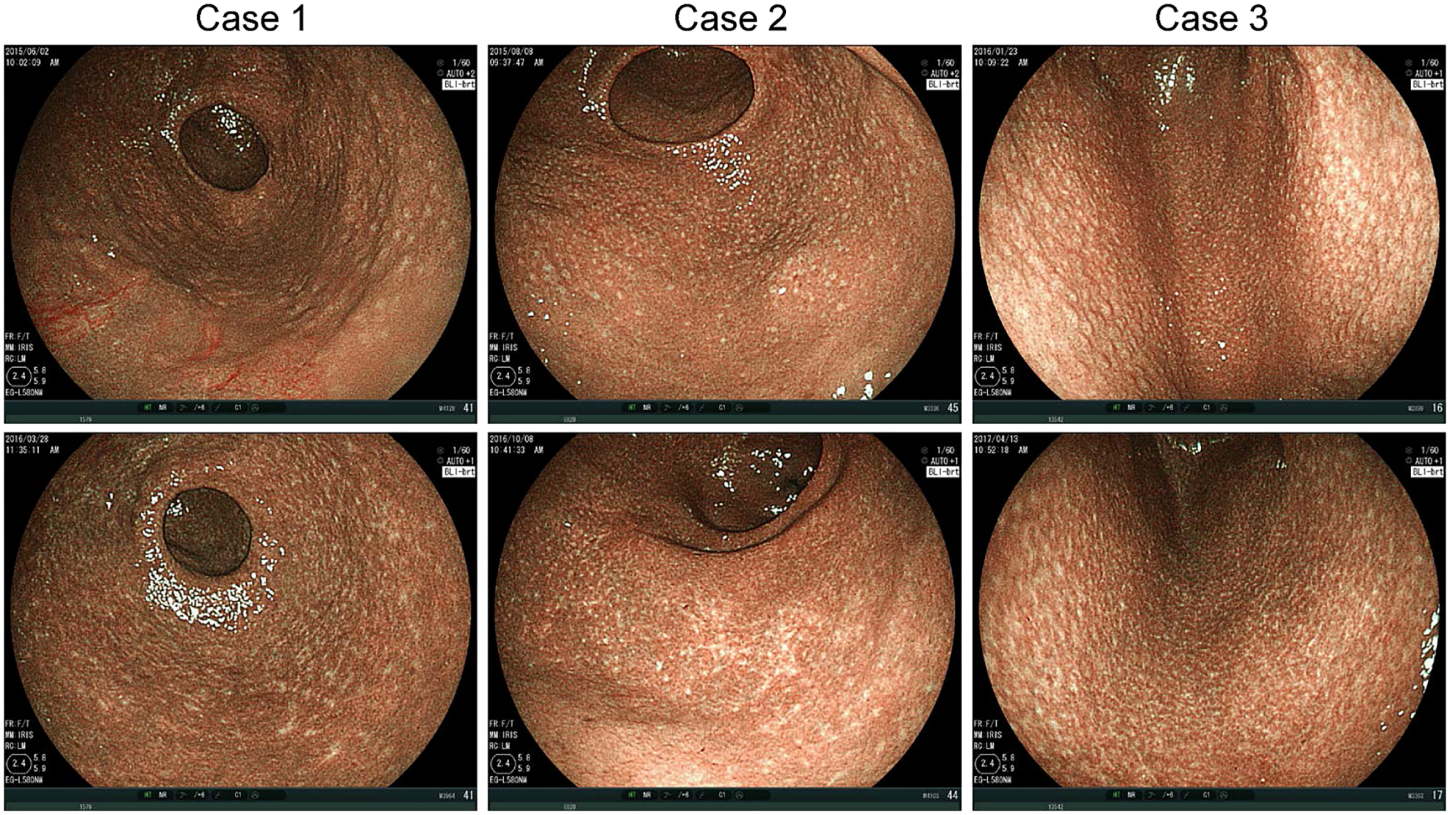
Change in atrophic mucosal pattern after HP eradication in patients with Spotty pattern. Upper panels: Images before eradication. Lower panels: Images taken 9 (Case 1), 13 (Case 2), and 14 (Case 3) months after eradication. HP, *Helicobacter pylori*.

Of the enrolled patients, 22 underwent HP eradication after the initial endoscopy and subsequent post-eradication endoscopy. The mean interval from HP eradication to post-eradication endoscopy was 12.8 ± 2.7 months. In all 13 patients with the Spotty pattern before HP eradication who underwent subsequent post-eradication endoscopy, the Spotty pattern disappeared and the Cracked pattern was detected (Fig 6). Of the 8 patients classified as having the Mottled pattern before eradication, the Mottled pattern persisted in 5, and 3 were newly found to have the Cracked pattern at the atrophic border on the greater curvature side. The one remaining patient was classified as having the Cracked pattern both before and after HP eradication therapy.

## Discussion

Image enhanced endoscopy has been extensively studied in relation to magnifying endoscopy, but its usefulness in intermediate and distant views has not been well established. Observation of atrophic gastritis by BLI in an intermediate to distant view enables endoscopists to obtain more detailed analyses and to classify mucosal patterns based on clear visualization of the patterns that cannot easily be seen under routine white light.

The present study included patients diagnosed as having atrophic gastritis based on white-light endoscopic findings. The current classification of atrophic mucosal patterns is based on endoscopic findings under BLI. In clinical practice, if any endoscopic finding of atrophy is detected during white light endoscopy, it can be expected that the pattern classification will immediately be confirmed by switching the observation system to BLI, using the current system. It is thus justifiable that the patients included in this study were those diagnosed as having atrophic gastritis based on endoscopic, rather than histological, features.

Classifying atrophic mucosal patterns observed by BLI is important for understanding HP-related gastritis. In this study, we defined three characteristic atrophic mucosal patterns observed by BLI: Spotty, Cracked and Mottled. The Mottled pattern includes a wide range of features not classifiable as either of other two patterns. This is an original classification developed by the first author, taking suggestions of other authoritative endoscopists into account. Therefore, this was the first experience with this classification for the endoscopists participating in the current study.

The Spotty pattern tended to be more common in patients who tested positive for HP infection and in younger patients. Although sensitivity was low, the specificity, positive predictive value and positive likelihood ratio for the diagnosis of HP infection based on the Spotty pattern observed by endoscopy were high, at 95.3%, 86.5% and 8.9, respectively. This raises the possibility that patients with the Spotty pattern are likely to have HP infection, although not all HP-positive patients have the Spotty pattern and not all patients without the Spotty pattern are HP negative. Given the relatively high agreement rate among interpreting endoscopists, the Spotty pattern is likely to be easily identifiable, and may, therefore, be useful for definitive diagnosis of HP infection.

The Spotty pattern appears to indicate recent HP infection, with subsequent gastritis at the infection site, and thus tends to be observed at the proximal end of a proximally extending HP-related gastritis. However, in our experience, it was considered that, even if the Spotty pattern is not present at the proximal end, the detection of the Spotty pattern in any part of the stomach may indicate the presence of active HP-related gastritis.

**Fig 7:**
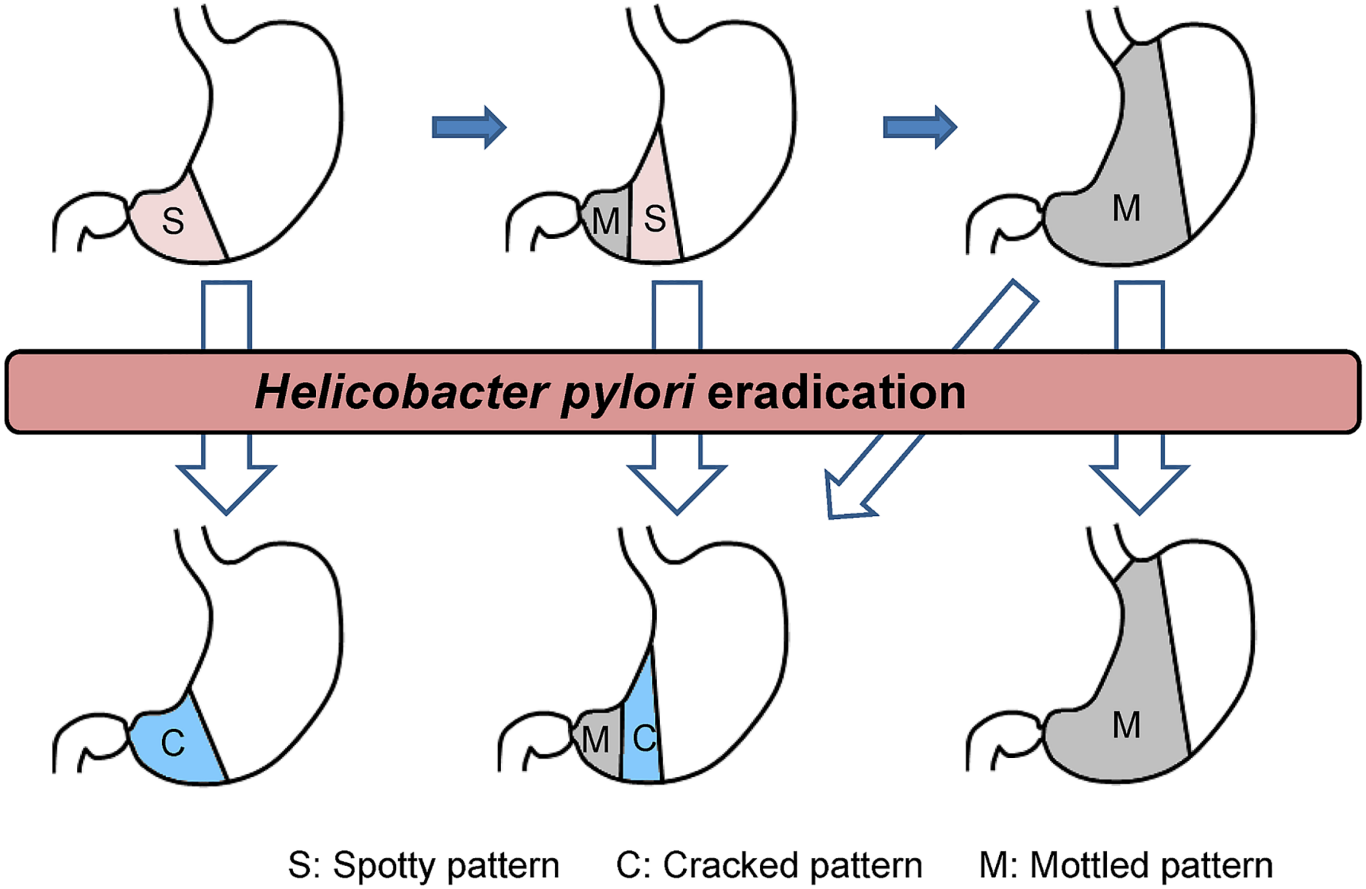
Changes in atrophic mucosal patterns associated with progression of atrophic gastritis and HP eradication. S, Spotty pattern; C, Cracked pattern; M, Mottled pattern.

Nodular gastritis [15], which was found in 9 patients in this study, presents with the Spotty pattern, but the findings are rather nonspecific and do not produce a typical clear Spotty pattern. When observed employing dispersed indigocarmine, the spotty areas of nodular gastritis are usually elevated, while in those 80 patients who were found to be free of nodular gastritis and classified as having the Spotty pattern in the current study, the spotty areas were flat or slightly depressed. This means that nodular gastritis is not equivalent to the Spotty pattern, instead comprising only a part of it. Among the patients who underwent biopsy, those with the Spotty pattern were likely to have lymphoid follicles as seen in patients with nodular gastritis (Table 2), suggesting that the Spotty pattern, like nodular gastritis, represents a change that is observed in an early stage of HP infection. Whether the elevated and depressed Spotty patterns represent different stages of the same disease, or different diseases, is an issue that remains to be addressed. Meanwhile, it appears that patients with more advanced atrophic gastritis are more likely to have the Mottled pattern and less likely to have the Spotty pattern (Fig 7).

**Table 2:** Histopathological findings in patients with each pattern.

	Spotty pattern	Cracked pattern	Mottled pattern
**Lymphoid follicle, Yes/Total**	13/20	1/12	0/28
**Intestinal metaplasia, Yes/Total**	4/20	1/12	20/28

The Cracked pattern was most common in patients who tested negative for HP infection, especially those who had undergone bacterial eradication, and the patients with Cracked pattern were older than those with Spotty pattern and younger than those with Mottled pattern. The Cracked pattern tended to be more frequently observed on the anal side of the atrophic border in the greater curvature of the gastric antrum. This issue warrants further study. The observation that the Cracked pattern was especially prevalent in HP-negative patients who had undergone bacterial eradication suggests that this pattern represents a healing process of HP-related gastritis. Some patients who had not undergone HP eradication tested negative for HP infection, and possible reasons include spontaneous eradication after antibiotic therapy for other diseases and spontaneous clearance of HP infection after adulthood.

If the Cracked pattern represents a repair process of gastritis, this pattern may also reflect mucosal repair in HP-positive patients who have experienced repeated resolution and exacerbation of HP-related gastritis. Unlike the Spotty pattern, the Cracked pattern does not indicate the presence of HP, but may indicate evidence of gastritis repair. This should be kept in mind when evaluating atrophic mucosal patterns.

In the present study, all 13 patients with the Spotty pattern before HP eradication had the Cracked pattern on post-eradication endoscopy (Fig 6). This would appear to suggest that HP eradication results in a change from the Spotty to the Cracked pattern. In fact, however, it seems to indicate the disappearance of the Spotty pattern which can in turn be taken as evidence of the clearance of HP, while a newly-appearing Cracked pattern is evidence of the repair process of gastritis. In elderly patients, for example, observation of the Cracked pattern in the gastric antrum and the Spotty pattern in the gastric body suggests the shift of a new infection site toward the oral side with repeated resolution and exacerbation of HP-related gastritis. Such a finding can be interpreted as indicating that, despite ongoing HP infection, the Cracked pattern was observed in a part of the stomach indicating prior repair processes. One of the important indications of the Cracked pattern would appear to be the possibility of determining whether a patient had never experienced HP infection versus having had such an infection but recovered from it. This would be especially useful for screening endoscopy. A patient without inflammatory findings but having the Cracked pattern in the gastric mucosa might be considered to have experienced HP infection in the past. This information is quite important for follow up observation of the patient.

As for the Mottled pattern, the white areas identified by biopsy generally contained intestinal metaplasia (Table 2), suggesting that the Mottled pattern represents intestinal metaplasia. When observed in an intermediate or distant view, the white portion of a light blue crest, which represents intestinal metaplasia on magnifying endoscopy, may correspond to the white portion of the Mottled pattern, reflecting also the histological structure of intestinal metaplasia. The fact that the Mottled pattern tended to be observed in the terminal stage of HP-related gastritis and was more prevalent in elderly patients suggests that this pattern reflects a long-lasting inflammation due to HP infection. While some of the patients classified as having the Mottled pattern before HP eradication may present the Cracked pattern in a portion of the gastric mucosa after eradication, the Mottled pattern tends to persist after HP eradication.

This study was based on the results of image interpretations by three expert endoscopists. Meanwhile, we also asked three non-expert endoscopists to interpret the same images, and noted that the kappa coefficient for the agreement rates between the experts and non-experts was 0.74, indicating a substantially high agreement rate. This result suggests that the current mucosal pattern classification can be made even by non-expert endoscopists, and thus may be widely adopted for the diagnosis of HP infection.

This study has limitations. First, histopathological assessment was carried out in only a limited number of patients, because we used biopsy samples collected from patients who underwent biopsy for diagnosis of HP infection. Detailed histopathological investigation is necessary in a future study. Second, the agreement rate on interpretation of endoscopic findings by the three endoscopists, as analyzed employing the kappa statistic, was insufficient. This is likely attributable to this being their first experience in interpreting such endoscopic findings. Further experience in this field would enable endoscopists to achieve a higher agreement rate in the future.

In conclusion, the Spotty pattern may indicate the presence of HP infection. The Cracked pattern may represent a post-inflammatory change as seen after HP eradication and may lead to determination of whether HP infection, now resolved, was present in the past. The Mottled pattern may represent intestinal metaplasia resulting from progression of HP-related gastritis. The classification of atrophic mucosal patterns applied herein should therefore facilitate the endoscopic diagnosis of HP-related gastritis. Since HP eradication may result in disappearance of the Spotty pattern and appearance of the Cracked pattern, further follow-up examinations to detect the changes in atrophic mucosal patterns after HP eradication will enhance the utility of this pattern classification as a tool for understanding the dynamic pathology of HP-related gastritis (Fig 7). Given the substantial agreement rates among interpreting endoscopists, the Spotty pattern is a potentially useful marker for HP infection. Further investigations are needed for the other two patterns, since their significance was not fully demonstrated in the present study. We anticipate that further histological studies will contribute to elucidation of the mechanisms by which each of these atrophic mucosal patterns is formed.
